# The Costs of Customer Service Citizenship Behaviors: A Qualitative Study

**DOI:** 10.3389/fpsyg.2020.00460

**Published:** 2020-03-17

**Authors:** Einat Lavee, Shani Pindek

**Affiliations:** Department of Human Services, University of Haifa, Haifa, Israel

**Keywords:** customer service, citizenship behavior, OCBC, OCB costs, informal resources

## Abstract

Organizational citizenship behaviors (OCBs) – behaviors not formally required or rewarded by the organization, but which promote its effectiveness – can be directed at coworkers, the organization itself or other stakeholders. OCBs directed at customers (customer-oriented citizenship behavior or OCBC) have received surprisingly little attention. Preliminary studies examined the unique contribution that OCBCs make in terms of perceived service quality and customer loyalty. In the current study, OCBCs were conceptualized in terms of supplying informal resources, which are– resources provided outside the worker’s formal role or the organization’s formal goals, or formal resources offered in informal ways (after hours, off duty). Applying a qualitative design, we uncovered types of informal resources and their associated costs. We also examined differences in informal resource provision and costs between occupational groups. Participants were 214 Israeli professionals who offer customer service in the education, health and welfare domains. All participants reported investing informal resources in their customers. Data demonstrated a remarkable range of types of informal resources, including emotional, instrumental and material resources. The most prevalent associated costs were interference with workers’ family life, followed by such personal costs as loss of free time, physical and emotional exhaustion, and material expenditures. Professional costs, which were rarely reported, included time taken from other customers and reduced in-role performance. No differences were detected in the pattern of informal resources between occupational groups: all employees reported high informal investment aimed at increasing customers’ well-being. Some of the implications discussed include the potential organizational costs associated with OCBCs. While such behaviors can improve service quality ratings, they can also lead to depleted employee resources and higher strain, negatively impacting productivity in the long term.

## Introduction

Organizational citizenship behaviors (OCBs) refer to employee behaviors that support the social and psychological work environment and promote organizational effectiveness. These behaviors can be directed at other employees, the organization as a whole or even the clientele. While considerable research has been devoted to uncovering the costs associated with OCB directed at other employees or the organization itself (see Bolino et al., 2013), little is known about costs associated with OCB oriented toward service recipients (customers, clients, patients, students and the like). The main purpose of the current research is therefore to uncover what types of efforts (resources) are invested in service recipients, as well as their associated costs.

Citizenship behaviors in organizations have largely been examined by quantitative designs, providing a consistent account of various factors involved in OCB, such as those that predict such behaviors (e.g., Eatough et al., 2011) and implications of OCBs for both employees and organizations (e.g., Hoffman et al., 2007; Nielsen et al., 2009). These studies, however, do not capture the individual experience of employees engaged in such practices, nor do they allow a more in-depth examination of the impact on the workers’ personal lives. We thus chose to apply a qualitative research method, which allows us to study the experience of employees, as well as to provide a more detailed and nuanced account not only of the scope of OCBs oriented toward service recipients, but also of their effect on employees’ work and non-work lives.

It should be noted that much of the existing OCB research refers to industries that cater to customers, whereas the current study involves public service professions, which mainly have clients, patients, or students. We will therefore use these terms interchangeably.

### OCB Conceptualization

Organizational citizenship behaviors were originally defined as discretionary behaviors that are not part of the formal requirements or reward system of the organization, but that promote organizational effectiveness (Organ, 1988). Early work on OCB focused on behaviors that benefit other individuals (OCBI: e.g., helping others who have been absent or who have heavy workloads) or the organization generally (OCBO: e.g., adhering to informal rules; giving advance notice when unable to come to work) (Williams and Anderson, 1991). These two types of OCB seem to have different underlying motivations, and while all motivations are linked to all types of OCB, status striving seems to be more closely related to OCBO whereas communion striving is related more to OCBI (Halbesleben and Bowler, 2007). Although these OCB dimensions exhibit high intercorrelations, they have some different predictors (e.g., Ma and Qu, 2011), different associations with role identities (Dávila and Finkelstein, 2013), and can have different outcomes, or different processes leading to those outcomes (Podsakoff et al., 2014).

As the study of OCB progressed, additional targets of these helpful behaviors came into focus. An important driving force in service organizations is the service recipient, such that citizenship behaviors oriented at customers (OCBCs) have become a topic of interest (e.g., Groth, 2005; Ma and Qu, 2011). OCBCs are defined as a unique form of OCB, with unique predictors and with the potential to foster customer loyalty and enhance perceived customer service quality. These behaviors, to name a few, can include being exceptionally courteous to customers, providing service quickly and with extreme care, speaking up for customers, and getting involved in issues that affect them (Ma and Qu, 2011; Lam and Mayer, 2014). In line with the general definition of OCBs, OCBCs are service-related actions that go beyond what the job requires formally. Thus, whereas providing adequate or even good service would generally be considered part of the job description, OCBCs are those behaviors that go “beyond the call of duty.” Seeing as different types of OCB have different underlying motivations, it is reasonable to assume unique underlying motivations for OCBCs as well. Specifically, while OCBO and OCBI are linked to status and communion striving (respectively), OCBCs might have altogether different motivations. For example, it might be egalitarianism (Piff et al., 2010) that is driving service providers to offer additional help to customers who are in need of greater assistance than what is formally provided within the service encounter.

Some OCBC research has focused on antecedents of these behaviors. Examples include some unique predictors, such as service climate (Schneider et al., 2005; Lam and Mayer, 2014), as well as predictors that are shared with other types of OCB, such as organizational commitment (Paulin et al., 2006; Rank et al., 2007), job autonomy (Lam and Mayer, 2014) and employee well-being at work (specifically, low burnout and high engagement) (Moliner et al., 2008). Other research has focused on the benefits of these behaviors, which have been linked to customer satisfaction and then to sales in a sample of 56 supermarket departments (Schneider et al., 2005), and to customer loyalty in a sample of medical sales representatives (Jain et al., 2012). In a field quasi experiment, Hui et al. (2001) found that training employees for OCBCs resulted in higher customer satisfaction with the quality of the received service.

### The Dark Side of OCB

Regardless of the target of the behavior, OCBs have mainly been studied as positive behaviors, motivated by prosociality (Bolino and Grant, 2016) and benefiting the organization and its employees. Examples of these benefits include receiving reciprocal help (Lyons and Scott, 2012) and getting better performance appraisals from supervisors (Whiting et al., 2008). However, there is now a substantial line of research uncovering the significant costs associated with OCBs (Bolino and Turnley, 2005; Bolino et al., 2013). For instance, engaging in OCB has been shown to interfere with perceptions of work goal progress, at least for some employees (Koopman et al., 2015). Researchers have argued that the costs associated with engaging in OCB, in terms of time and energy, often outweigh the benefits, and that resources invested in helping others are being allocated away from employees’ sanctioned task performance, which can potentially damage their career success (Bergeron, 2007). OCBs have a cost for the employee in terms of increased workload, general job stress, and even work–family conflict (Bolino and Turnley, 2005), and they are believed to diminish one’s ability to be active in one’s community (Kark and Waismel-Manor, 2005). OCBs can also hurt employees’ job satisfaction, especially at very high levels and for individuals low in optimism (Munyon et al., 2009). OCBs can even have negative effects on group-level job performance, particularly when the task does not entail much interdependency between coworkers (Nielsen et al., 2010). While there is considerable research showing there are negative consequences for the general forms of OCB, the current study is novel in examining the dark side of one specific type of OCB, namely, customer-oriented OCBs (OCBCs). The purpose of the current study is therefore to portray the complexity of OCBCs using three research questions addressing the types of resources, the associated costs, and differences between occupational groups.

### Theoretical Framework and Research Questions

When employees provide their customers with help that goes beyond the call of duty (OCBCs), they are investing their personal time, energy and other resources in those customers. We label these resources as informal resources, because while some resources are sanctioned by the organization and are invested in customers as a formal requirement of the job (e.g., service encounter time), these sanctioned resources often do not suffice to meet the needs of the clientele (Lavee and Strier, 2019). Therefore, informal resources refer to the resources themselves (e.g., personal time) and OCBC refers to the investment of these resources in customers (e.g., spending personal time after work hours with a client).

Gleaning from the public administration literature, the reduction of organizational resources is increasingly prevalent (Brodkin, 2011). In other sectors, the always growing need to remain competitive results in needing to “do more with less” (e.g., Simons and Mason, 2003) and this includes cuts to service production. One example is converting portions of the service encounter into self-service technology, thereby reducing the paid employee time resource that is invested in customer encounters. However, this reduced paid time, resulting from the need to cut costs, shifts part of the responsibility for producing quality service to the customer (Hilton, 2013). When costs are cut but solutions (e.g., technological solutions) are not provided by the organization, the responsibility remains with service providers, who then have to “do more with less” on their own.

When sanctioned resources are insufficient, employees may choose to provide additional resources that go beyond what the organization offers formally, particularly to customers they perceive as being in greater need (Lavee et al., 2018). Those resources are given discretionally and considered informal resources. Providing customers with those informal resources constitutes OCBCs, because these are employee behaviors aimed at helping customers beyond what is formally required by the organization, and at the expense of the employee. Therefore, in terms of personal versus organizational resources (Xanthopoulou et al., 2007), our focus is on informal resources as a certain type of personal resource – one that can be transferred to customers (e.g., time, energy, money), as opposed to personal resources that cannot be directly transferred (e.g., self-efficacy). Little is known about the scope of service employees providing informal resources to customers or what those informal resources encompass. Therefore, the first research question in the current study is about the types of informal resources provided.

Research Question 1: What informal resources are provided by customer service employees and how prevalent is this phenomenon?

Investing informal resources incurs a cost taken from the employee’s own resource pool. This process can be understood via the lens of the Conservation of Resources (COR) theory (Hobfoll, 1989; Hobfoll et al., 2018), which posits that stress occurs when important resources are threatened with loss, are actually lost, or there is failure to gain further resources after a significant effort (resource investment) is made. The actual investment of informal resources, that is an inherent part of OCBCs, includes a loss of resources for the employee. If these efforts are not recognized or rewarded in any way, then it is likely that no further resources are gained following this investment of time, effort and other resources. This imbalance between informal resources that were invested (and therefore lost) and the lack of resource gain results in resource depletion or resource drain (Rothbard, 2001). In other words, individuals have a finite amount of physical and psychological resources, and tradeoffs are made between the different roles that require the individual’s resource investment.

This is related to the conceptualization of role conflict, whereby demands from one role deplete available resources, making it harder to meet the demands of another role (Greenhaus and Beutell, 1985). For example, time-based conflict is the idea that time is a finite resource, and when time is spent meeting demands from one role, there is less remaining time available to meet the demands of other roles. Another example is strain-based conflict, whereby the strain experienced because of the resource drain in one role makes it difficult to fulfill the requirements of another role. There is evidence that when employees are overly engaged, they perform more OCBs, and those OCBs deplete resources in a way that interferes with other roles they hold (Halbesleben et al., 2009). These include their family roles, but can also such roles as their personal relationships in and outside of work, their hobbies, and the like. Role conflict also exists within the work domain, when an employee experiences conflicting demands (Rizzo et al., 1970). Specifically, if the demands that arise from providing service to one customer are too great, such that informal resources are invested, it is possible that fewer resources (e.g., time, energy) remain available for other customers.

In the context of OCBCs, such efforts (i.e., the investment of informal resources) may go unnoticed or unrewarded because “doing more with less” is the general rule of conduct in organizations, and may also be less observable than OCBs directed toward other employees (OCBIs) or toward the organization as a whole (OCBO). This is because the specific content of the interaction with customers, where informal resources are provided, is usually not monitored but rather only the outcome of the service interaction is noted. Therefore, customer service employees may provide informal resources without receiving any acknowledgment or rewards for their efforts. According to COR theory (Hobfoll, 1989; Hobfoll et al., 2018), this investment of resources, which does not hold adequate potential for future resource gain, constitutes resource depletion, which has costs in terms of well-being and functioning in both work and non-work domains. Though research has examined costs associated with general OCB (e.g., Bolino and Turnley, 2005; Bergeron, 2007; Bolino et al., 2013), an important question that remains unanswered focuses on the costs associated with OCBCs.

Research Question 2: What are the personal and professional costs of engaging in customer service citizenship behaviors (OCBCs)?

In addition to mapping out the costs associated with OCBCs, it is also important to examine differences between occupational groups. Specifically, working conditions (e.g., workload, work stress, job resources, salaries) vary extensively between occupations and occupational groups. Another issue that may be relevant is the level of neediness of the customer, where some occupational groups provide services primarily to impoverished clients (e.g., social workers) while others encounter clients from all walks of life (e.g., medical doctors). While the current study could not include all occupations that offer customer service, we were able to compare three occupational groups. We focus in the current study mostly on professional service providers, i.e., providers whose profession typically requires complex skills and higher education, as opposed to non-professional employees who provide customer service as their occupation (Thakor and Kumar, 2000). We therefore pose an additional research question regarding differences in the investment of resources and associated costs between different occupational groups.

Research Question 3: Are there differences in the types of informal resources and costs among different occupational groups?

## Materials and Methods

This article is part of a larger study which examines interactions between front-line workers and their clients. Under the assumption that front-line workers often provide clients with resources beyond the formal requirements, we chose the qualitative-constructivist research method as a means to delve into this phenomenon, as it allows for in-depth understanding of individual experiences, perceptions, decision-making processes and personal beliefs. A qualitative, inductive method, closely tied to grounded theory (Glaser and Strauss, 1967), was employed in an attempt to uncover the dynamics of workers’ OCBC, as well as the possible costs associated with them. This method enables us to understand processes in their natural settings and interpret phenomena in terms of the meanings people assign to them (Denzin and Lincoln, 2011).

### Participants

We sampled employees from three occupational groups: education, health care and welfare. These groups were selected because they suffer a continuous decrease in organizational resources, forcing employees to “do more with less.” This is true both in Israel (Maron, 2015) and elsewhere (Hupe and Buffat, 2014). We chose not to focus on a single occupational group, but rather included several, in an attempt to yield a rich and refined portrayal of OCBCs that considers variations related to each profession and job setting.

The study draws on the accounts of 214 Israeli service employees. Most participants were front-line workers; a few were higher-level workers. All participants provide customer service as part of their jobs. Participants from all occupational groups were mostly professionals, although some non-professionals were also included. The education group (*N* = 65) included teachers, as well as other school workers, such as the administrative staff. The health care group (*N* = 56) included doctors and nurses, other professional workers such as physiotherapists and other therapists, and other workers such as clinic secretaries. The welfare group (*N* = 93) included social workers, and other workers such as employment consultants, supportive housing workers and welfare administrative staff. The majority of the participants were women (*N* = 152, 71%). This gender distribution is consistent with the general overrepresentation of women in social service occupations, specifically social workers, nurses and teachers (United States Department of Labor, 2018). The gender distribution for each occupation is presented in Table 1. Participants ranged in age from 20 to 72, with an average of 40.5 (*SD* = 11.6). Most were married (*N* = 146, 68%); others were single (*N* = 23, 11%), divorced (*N* = 11, 5%), widowed (*N* = 3, 1%) or declined to reveal their family status (*N* = 35, 16%). In terms of ethnic distribution, 166 participants were Jewish (78%) and 48 were Arabs (22%) – most of them Muslim, with a few Christians and Druze.

**TABLE 1:** Gender distribution within occupations in the sample.

Welfare	Female	Male	Total
Social worker	52	3	55
Department manager	2	2	4
Neighborhood worker	2	0	2
Hostel consultant	3	0	3
Psychologist	0	2	2
Employment consultant	9	1	10
Rehabilitation consultant	5	0	5
Other	10	2	12
Total for welfare	83	10	93
**Health**			
Doctor	9	20	29
Nurse	20	2	22
Others	5	0	5
Total for health	16	40	56
**Education**			
Pre-school teacher	2	0	2
Teacher	48	12	60
Other	3	0	3
Total for education	53	12	65

We recruited interviewees based on theoretical sampling (Warren, 2001), which allowed us to seek out participants who seemed likely to epitomize the analytical criteria in which we were interested. Specifically, we made contact with key actors in all three occupational groups who then referred us to additional potential participants. Inclusion criteria included working currently in one of the three occupational groups mentioned above, and having direct and daily interaction with service recipients. According to the grounded theory approach, this type of sampling aids in developing the study’s categories or theory, and is not meant as a representative sample of a particular population (Bryant and Charmaz, 2012).

### Measures

The research tool was a semi-structured in-depth interview. The interview protocol was designed to provide comprehensive evidence of the everyday experience of providing customer service, with a specific emphasis on informal resources. Workers were first asked to describe their general work routine and their formal job requirements. They were then asked to describe the resources they provide to clients. The main part of the protocol focused on providing informal resources: workers were asked to describe how they provide such resources to clients, if at all. After the interviewers clarified the distinction between formal and informal resources, respondents were asked to describe the types of informal resources they provided to clients. Finally, workers were asked to describe the costs of providing such resources.

### Procedure

Interviews were conducted either by the authors or by trained research assistants. All interviews were conducted face to face, mainly in the interviewees’ workplace, which was their preference. Others were conducted in coffee shops or participants’ homes. Anonymity and confidentiality were ensured. All interviews were audio recorded, transcribed and analyzed. No compensation was provided for the informants’ time.

### Data Analysis

All data analyses were conducted using the Atlas.ti software. While the general analysis was part of a broader study examining interactions between front-line workers and their clients, in this paper we focused only on the codes relevant to our research questions: types of informal resources, costs for workers and occupations.

As with most qualitative analyses, the process was iterative and involved switching between data, codes and emerging schemes (Charmaz, 2014). The data collection and analyses were approached without *a priori* assumptions about participants’ perceptions and actions. Data was coded by two research assistants and one of the authors. The coders were trained by the author, who has rich experience conducting qualitative studies and training research assistants in the coding task. The author coded several preliminary transcripts and created an initial coding scheme. Then the first research assistant coded all the data, and continued developing and creating new codes as they emerged from the data. This process was conducted under continuous consultation with the author. Finally, the second research assistant reviewed the data and checked the coding. When disagreement with the first research assistant emerged, it was resolved in a discussion with the author.

Following grounded theory procedure for data analysis (Charmaz, 2014), the first step was open coding. In this step, we categorized each piece of the data with a short name (i.e., code), and created initial categories (which codes belong together) that would later be refined. However, in contrast to other studies, wherein it is relatively easy to divide and separate different codes, in the current analysis we confronted an analytical difficulty embedded in the nature of both resources and costs, many of which could be coded under several categories. For example, the informal resource of “giving rides in personal car” could be seen as a material resource, since it saves money for clients that they would otherwise have to spend on public transportation. It could also be categorized as a time investment, or as emotional support, part of extra personal treatment. The words of a hospital nurse demonstrate how these types of resources are interlinked:

They just didn’t understand what they need to do, neither in Russian or Hebrew. So I translated for them what clinic they need to go to, where and when. But then I saw they were completely lost and needed more support, so I told them, “Don’t worry, I’m with you,” and I made some phone calls, escorted them to the clinic they needed to go to, made sure they reached the right place.

Similarly, in terms of costs to workers, “giving rides in personal car” could be categorized as either a material cost (money for gas) or as time spent off duty with a client which would otherwise have been spent on personal matters. For the sake of analytical clarity, we present both resources and costs divided by types, but it is important to note that they often overlap.

The second step was axial coding, i.e., refining the code categories in a way that represents commonalities among codes. According to Strauss and Corbin (1990), this reflects the idea that codes can be clustered around specific axes. For example, segments such as:

I pay a heavy personal price. I see my kid less, spend less [time] with my husband. Even on weekends I come to work to close gaps. It’s not normal and the family doesn’t always understand and support [me], and it creates arguments and bad feelings because of work.

were categorized as *personal cost/impact on family*. Conversely, segments such as “It takes away my peace of mind. Makes me think, I’m not really calm when I’m home and I’m busy with work-related things.” were categorized under *personal cost/emotional burden.* One example of the iterative nature of the coding is that only after coding all the segments did we realize that *impact on family* differs from other personal costs and thus should constitute a separate category.

The third and final step was selective theoretical coding. At this stage, the categories and their interrelationships were combined to form a storyline that addresses the research questions. This form of analysis enables the conceptualization of an analytical story and its coherent presentation.

## Findings

### Research Question 1: Types of Informal Resources

The first research question examines the prevalence and manifestations of employees’ engagement in OCBCs – providing their clients with help beyond the call of duty. We did so by focusing on the informal resources employees gave their clients. The analysis revealed that employees from all three occupational groups reported that they provided informal resources to clients. We found a remarkable range of informal resources. Table 2 presents the four types of informal resources provided by employees, divided by type of resource (material, emotional, instrumental and time investment).

**TABLE 2:** Informal resources provided by workers, by type of resource.

**Material resources**
Cash
Groceries
Medicine
Clothing and shoes
School materials
Giving rides in personal car
Tickets for sport games
**Emotional resources**
Emotional and psychological support
Hosting clients in worker’s home and family
Extra personal treatment
Motivational talks
**Instrumental resources**
Providing information not related to formal duties
Assisting with bureaucracy
Translating formal forms
Training and consulting
**Time investment**
Home visits
Providing services to clients’ family
Giving private phone number
Investing extra time in clients
Thinking about clients while off duty
Staying with clients after hours
Staying after hours
Assisting with issues not included in formal work duties
Accompanying clients in handling bureaucratic errands
Brokering – recruiting resources from others (including material resources)

#### Material Resources

Material resources included cash or other material products. A third-grade teacher explained:

I have many examples. There is one day that the students eat in the school cafeteria. All the children went to eat and one kid stayed in class. After talking with him, I realized that his family could not afford to give him money. Without thinking twice, I gave him the money (interview 9).

Others reported buying groceries: “Sometimes when I make a home visit and see they have absolutely nothing in the refrigerator, I go to the grocery store and leave food packages outside their door” (social worker, interview 13). Another common in-kind material resource is money for public transportation: “If I know someone could not afford the bus ticket he needs to go to the doctor’s office, or to the welfare office to receive the rights he deserves, I pay for the bus ticket” (social worker, interview 16). In the absence of formal organizational resources, the workers personally provide these material resources, which are often given when the worker perceives the situation as an emergency.

#### Emotional Resources

The second type of informal resource was emotional. Workers reported providing psychological support to clients or maintaining extra close relationships with them. Although in some professions, emotional and psychological support are inherent to the formal job, the participants made a clear distinction between formal and informal emotional resources they provide to clients.

For instance, a worker in the employment section of the welfare office stated:

I do give clients a lot of psychological support, it’s not professional or anything. But if I see they need it, I do it. Give them emotional support, listen to their problem. Then a meeting of an hour is spent empowering the client instead of employment training (interview 199).

Another aspect of emotional informal resource is extra personal treatment. For example: “Recently I escorted a client who is a police officer, who had severe problems at home, to the bank, to help him get the required forms so he can receive economic assistance from his workplace” (social worker, interview 2).

Some workers even provided emotional resources in the form of inviting clients to their home, which clearly goes beyond the job’s formal requirements: “There are students I invite to my house on Friday evening. I talk with them and give them some maternal feeling. This way they can get a sense of home, some warm feelings” (teacher, interview 81).

#### Instrumental Resources

The third type of informal resources was providing information, training and consulting beyond the requirements of formal duties. One participant, who works as an administrative assistant in a school, reported:

Some students can get scholarships, so the parents ask me to give them information about available scholarships, even though this isn’t part of my job. After that, I also help them fill out and submit the forms. And again, scholarships are not part of my responsibility (interview 51).

Similarly, workers often assist clients with bureaucratic red tape: “I help some clients appeal to government agencies, such as the National Insurance Institute. I do all this outside of my formal duties. I help them write letters, make phone calls and send faxes” (social worker, interview 104).

Another aspect of instrumental informal resources revealed as a major manifestation of OCBC involves translating formal forms. While Hebrew is the formal language in Israel, the society contains several minorities, such as Arabs and immigrants from the Former Soviet Union and Ethiopia. Clients belonging to these minorities often struggle to understand the language in formal forms. Workers reported spending a large amount of their time translating for them. One Arab participant, a doctor in a local clinic, related: “The seniors, I try to explain to them in simple words. Often I translate the language, read them a certain letter or medical form” (interview 120). In addition, many workers who themselves are not part of the client’s minority group reported teaching themselves the language of their clients so they can better assist them. For example, workers who immigrated from the Former Soviet Union learned Arabic, while native Hebrew speakers learned Amharic (for Ethiopian clients).

#### Investment of Time

Beyond the specific resources outlined above, the analysis revealed extensive engagement of employees in OCBCs manifested in terms of time and availability. These informal resources included providing services for the client’s family; giving them one’s private phone number; staying with clients after hours; remaining after hours to complete required formal work they did not manage to finish because they had spent extra time with clients during formal working hours; assisting with issues not included in formal work duties; and thinking about clients while off duty. This general type of informal resources was expressed in quotes such as: “Often I stay after work hours, at my own expense, so clients can get an adequate response” (rehabilitation consultant, interview 32); “I invest a lot of time, more than I’m required by my work duties, and I’m also available after work hours” (social worker, interview 42).

Participants even gave clients their private phone number. This is a practice that is rarely formally required, but was often reported by interviewees. In the words of one social worker: “I think I even overdo it. Many of my clients have my phone number” (interview 34). Giving out one’s private number means working at all hours of the day. A youth consultant related: “Some kids text me at 6, 7, 8 pm, crying, asking if I can meet them. So I go out and meet them, calm them down. This is the classic case and it happens a lot” (interview 49). Similarly, a teacher explained:

I give them my number and always answer. If a kid needs me at 8 pm because something happened, or he didn’t understand the material, or he has a question, I’m still his teacher and I always answer the phone (interview 52).

Another aspect of time investment entails brokering, i.e., recruiting resources from others. Via such involvement with service workers, clients can receive resources that would not otherwise be available to them or that they would have to purchase themselves. One teacher reported:

There was a girl, I knew her family has financial problems. So I contacted City Hall and she received vouchers for lunch and tickets for public transportation. Later I found out their refrigerator was broken, so I called the welfare office and they took care of it. And then her mother told me she cannot set up a meeting with welfare, so I arranged the meeting with the social worker in the office of the school principal (interview 54).

Another example demonstrates the broad variety of resources brokered by workers. An employment consultant related:

My husband has three lawyers who work with him. Sometimes clients tell me they need some minor legal assistance, for example, a lawyer’s signature on formal documents, and it costs a lot of money. So I send them to one of these three lawyers and they do it for free (interview 198).

### Research Question 2: Costs Associated With OCBCs

The wide range of OCBCs described above result in a broad range of costs to employees. The analysis revealed various aspects of emotional, physical and material costs. These represent the “dark side” of customer service citizenship behaviors. We arrange these costs into three broad categories: familial, personal and professional (see Table 3).

**TABLE 3:** Costs of providing IFRs to clients.

**Familial**
Availability for children
Relationships with partners
**Personal**
Reduced free time
Physical fatigue
Emotional burden
Material/economic costs
**Professional**
Time taken from other clients
Reduced in-role performance

#### Impact on Family

The most prevalent costs associated with OCBCs were the impact on employees’ family life, their partners and children. This was mostly due to the extra time devoted to clients and availability, as is clear from the words of a male teacher: “The costs are massive. All this time and energy that I spend beyond the formal requirements of my job. My family is the first to suffer” (interview 82). The understanding that engaging in OCBC activities has far-reaching consequences for family is discernible in most of the interviews, regardless of the gender of the service provider.

One rehabilitation consultant reported the impact on her availability to her children: “As for personal costs, you have to ask my kids [laughing]. Sometimes they say that I am more devoted to work than to them” (interview 4). This devotion to work hurts both the worker-parent and his or her children. However, the interviewees made a distinction between personal costs and costs to their children. In the words of one social worker:

I don’t feel that I paid a cost, but my kids were the ones who paid. I mean, their mother was absent. She wasn’t there. Instead of being with them in the afternoon or during holidays, I was busy looking for holiday donations to clients. I feel that they missed a lot, by me devoting myself to work at all costs and at any time. My kids definitely paid the price for that (interview 28).

Similarly, a teacher said: “I see the price, on my kids. I see their faces when the phone rings, and it’s really difficult. There are almost no evenings without phone calls during the afternoon or evening” (interview 75).

These responses show that the informal part of the job creates an added burden and increases work–family conflict beyond the effects of formal job requirements. Such conflict is apparent for both female and male employees, but has particular ramifications for women, who constitute the majority of social service providers.

Like the impact on children, interviewees reported costs affecting their relationships with partners. A nurse related:

It takes a lot of energy, it’s really exhausting. Every time I do something beyond [the call of duty], I give a part of myself. And when I come home I don’t have energy, I just want to sleep. At home, I just want to be in silence. My husband always asks, “Why don’t you talk with me? Why are you always tired?” I don’t even have the energy to argue with him (interview 131).

Along the same vein, a teacher reported:

Personal costs? No doubt! Instead of spending time with my husband, doing what normal people do, my thoughts are often focused on my students. My husband laughs and says he should be a student in my class, that way he could get more of my attention (interview 81).

#### Personal Costs

While the above costs – in terms of reduced time and energy for children and partners – had an indirect effect on the workers themselves and so can be considered personal costs, interviewees also reported costs that directly affect themselves. First and foremost, the analysis indicates the informal resource of *time investment in clients* to be the highest cost for workers. One aspect of such costs is a loss of free time outside of work. As one teacher explained it:

You always have to do extra, and your time is never enough. So I have to work extra hours from home, without pay of course. I try hard to regulate the cost so I can have free time, but usually without great success (interview 14).

The interviewees clearly acknowledge that they themselves pay a price for engaging in informal practices. A doctor said:

I pay a personal price because I spend my time on things beyond my job requirements. Today, for example, I sat with a patient on bureaucratic stuff, instead of doing other things. And then I will have to do these other things at night, at the expense of my personal time (interview 38).

Similarly, one social worker stated:

Most of my time is devoted to work. I just don’t have time to do other things. This is not because my work hours are so long, but because I just do more. This robs me of most of my leisure time (interview 42).

Another social worker related:

When I finish work and go home, I know that I’m not done. Many clients have my private phone number and call me at all hours. I can never relax, even in my own home, my mind is always busy with work (interview 49).

The above quotes indicate that, due to ongoing extra-role investment in clients, the boundaries between work and home become blurred.

Interviewees also pointed to an *absence of personal time during work hours*. Many reported that, due to their OCBCs, they have to skip breaks, meals and even going to the restroom. A nurse explained:

Often I know that when I overdo something for clients, for example, calming down a patient before surgery, it will affect my personal time. I can’t go to the restroom, won’t have coffee or lunch even though I’m hungry (interview 130).

Beyond the costs created by general time investment, the analysis also demonstrates personal costs of *physical exhaustion and emotional burden.* While these are two separate things, they were often described together. In the words of a psychologist: “The consequences of giving more than the formal work hours are being tired and having great fatigue, an intensity that robs me of my time, energy and attention from both home and myself” (interview 72). A teacher described it:

Sometimes you have to move mountains, and although I presumably do it by “choice,” when I come home I feel like I’ve been working in construction, completely empty. Emotionally, it’s extremely difficult. It takes a lot of emotional and physical resources (interview 22).

The literature commonly reflects reports of physical or emotional exhaustion from jobs in social services. What is of significance in the current findings is that the narratives emphasized exhaustion in relation to informal practices more so than to formal job requirements, not unlike the case of interviewees’ reflections on the work–family conflict presented above.

Sometimes investing informal resources can even cause physical harm. For example, when asked about the personal costs of OCBCs, a doctor said:

It’s time, emotional resources, as I told you it takes even more of the time that I have to devote to formal work, and it’s really stressful. It affected me very badly, I even had a stroke caused by all this stress (interview 120).

Finally, in light of the reports of material resources that workers provide to clients, we expected the interviewees to point to *economic costs.* Surprisingly, very few acknowledged that they paid a material price for providing material resources to clients, as demonstrated in the following:

Interviewee: I have cases that I take personally, for example, women who get divorced and find themselves in bad economic distress. In these cases, I search for donations and even buy them stuff so they can settle down in their new home, paying with my own money or bringing them stuff from home – blankets, electric heaters and more.

Interviewer: Do you think you pay a personal price for that?

Interviewee: Not really, only that I take it personally and it affects me emotionally (social worker, interview 200).

This finding is particularly interesting, as most participants were employed in jobs for which the salary is not high – teachers, social workers, nurses and doctors in the public sector. Notwithstanding the actual loss of personal economic resources, they did not perceive it as an actual cost. When asked directly about material costs, the most common response was to link the time devoted to informal resources practices and economic loss: “Many times it takes an economic investment, that no one pays me for, such as when I spend hours giving private lessons to students on my own time” (teacher, interview 82); “My material cost is to keep working more than my work hours, such as when I answer phone calls on my private time, no one pays you or rewards you in any way” (social worker, interview 43).

#### Professional Costs

The third type of cost associated with workers’ engagement in customer service citizenship behaviors was professional. The investment of informal resources in clients often hinders the workers’ ability to adequately meet their formal work requirements. This type of cost, however, was the least reported by participants.

One aspect of professional cost is *time taken from other clients*, as manifested in the words of a school administrator: “I totally acknowledge the fact that some clients are dissatisfied with me. There are clients who need extra service, and when you provide them with such service, you just don’t have time to invest in others” (interview 211).

A second aspect of professional costs is *reduced in-role performance*. This is a more general category that overlaps with the time taken from other clients. One social worker explained: “Sometime it hurts my professional outcomes, those that I’m being measured by. There are days when I take care of five people, when I actually have fifteen clients” (interview 76).

The cost of reduced in-role performance can also be manifested as reduced professionalism. Referring to OCBCs, one doctor said: “It’s a burden. It increases your work load, but also makes you deal with a lot of things beyond your professional skills, and sometimes you don’t have any knowledge of them” (interview 67). Another doctor explained: “Often I don’t do proper medicine. It comes at the expense of scholarship, of reading more about a patient’s condition, of advancing my own career-related stuff” (interview 210).

### Research Question 3: Similarities/Differences Between Occupations

The qualitative analysis revealed a general similarity between the occupations under study. The data revealed a great variety of informal resources in all three occupational groups, and they all reported costs associated with these practices. In an effort to provide a richer picture, and because qualitative methods do not easily lend themselves to quantitative analyses, we used cross-coding of the occupations with both informal resources and with costs, with the aim of distinguishing between combinations that were common and those that were not.

#### Differences Between Occupations in the Provision of Informal Resources

With respect to informal *material* resources, the analysis revealed that teachers are the main providers of this type of resource, with most of them reporting that they did so. Welfare employees reported supplying informal material resources less often, while health care employees reported the least frequent provision of this type of resource.

In contrast, virtually no difference was found between occupational groups with regard to informal *emotional* resources, even when focusing on the main aspect of giving personal treatment and support beyond the formal role. The only noteworthy exception was nurses, who reported providing this type of informal resource less, while also reporting provision of more personal attention and extra support to patients formally. It is possible that, in the nursing profession, this type of resource is considered an inherent part of the overall job, more so than for employees in other service professions, and therefore was not seen as informal by the nurses who were interviewed.

Informal *instrumental* resources include assistance with many issues that are not part of the formal role. The analysis revealed that teachers provided this type of resource to the greatest extent, followed by employees in the welfare occupations, and then nurses. Medical doctors were least likely to report provision of this resource.

The resource of *time* is divided into several categories, which vary in terms of the difference between occupations. The aspect of availability as an informal resource was widely reported by all employees, with most saying they are available to clients even outside of formal work hours. This is reflected, for example, in the large majority of employees reporting they give clients their personal phone number. Again, the professional position that was an exception to the rule was nurses, who did not mention availability as a widely provided informal resource. It is possible that the difference between professional positions is related to the needs of clients: most of the nurses interviewed worked in hospitals, and their availability was mostly required during the formal shift. This is in contrast to other health care professionals, such as community-based nurses or doctors, who broadly mentioned availability as an informal resource provided to patients. Finally, the resource of offering service and work for no extra pay, for a variety of aspects related to time investment, was widely mentioned by teachers, with the vast majority reporting they provide this informal resource. Working without pay was less frequently reported by welfare employees and in health care occupations (where there was virtually no mention of this resource).

#### Differences Between Occupations in Terms of Costs

Three types of costs were mentioned by interviewees: familial, personal and professional. Familial costs were frequently reported in all occupations, as employees in all professions felt that providing informal resources was detrimental to their families.

Regarding personal costs, costs related to physical fatigue and emotional burden were frequently mentioned by employees in education and welfare, particularly professionals, that is teachers and social workers. This cost was reported less often by health care employees, although it was still evident. Differences also emerged with respect to another type of personal cost, namely, economic costs, which were noted most often by teachers. This difference between occupations may be related to the above-mentioned high prevalence of teachers providing informal material resources, particularly when taking into account the low wages that they typically earn in Israel.

Finally, differences in professional costs were examined. Relative to other types of costs, these were mentioned less. Nonetheless, we did find differences between occupations in the context of time taken from other clients and reduced in-role performance: nurses mentioned this cost more than workers in other occupations. An explanation for this difference may lie in the nature of their work, as it may be more difficult for nurses to continue working when they are not physically present at the hospital. When they invest more in certain patients, it is harder for them to compensate using their personal time, such that it invariably results in impairment of formal functioning with the other patients. Lastly, the analysis revealed that medical doctors mentioned damage to career development and reduced professionalism within the service encounter (as demonstrated above), while other types of employees rarely mentioned such issues.

## Discussion

The main goal of this study was to examine the costs associated with OCBCs for service employees from different occupational locations. For that purpose, we first asked if workers indeed engage in OCBCs, focusing on the informal resources that they provide to service recipients. The data analysis revealed that workers indeed engage in high levels of OCBCs, providing a wide range of informal resources to their customers. These informal resources entail not only the investment of extra time and effort, but also the provision of material, emotional and instrumental resources. These results are in line with the notion that, as organizations struggle with the growing need to remain competitive and to reduce costs, the resources required for employees to adequately deliver service to customers might be reduced as well (Lavee and Strier, 2019). When no alternative solutions are offered by the organization, the responsibility remains with service providers, who then have to “do more with less” at their own expense. The data analysis demonstrated that this trend might be manifested in the form of informal resources.

We then examined whether the investment of informal resources might affect the service provider’s own resources, and thus focused on the costs of engaging in OCBCs. The analysis indeed revealed multiple costs for employees, in both work and non-work domains. The most substantial cost was found to be an impact on relationships with family. Additionally, the data revealed a depletion of personal resources in terms of a wide range of emotional, physical and economic costs. Lastly, providing informal resources also affects employees’ professional life, in terms of their ability to perform well in their formal role and to advance their career, though to a lesser extent than family and personal costs. The large scale of costs for employees, which is aligned with COR theory (Hobfoll, 1989; Hobfoll et al., 2018), points to a wide gap between the workers’ investment of resources and adequate potential for future resource gain, leaving employees depleted of resources.

Interestingly, participants who provided monetary support to their customers did not perceive the loss of personal funds as a cost, but rather highlighted other informal resources that were invested (e.g., time, energy). When asked about this practice, very few interviewees acknowledged that they paid a material price for supplying material resources to clients. One possible explanation is rooted in social comparison theory (Festinger, 1954), according to which individuals evaluate their own worth based on comparisons with others who are faring better or worse. In the described OCBC, customers were in a particularly bad financial state. This could lead service providers to feel that, relatively speaking, their own situation is not bad. In that sense, providing this particular informal resource (i.e., money) may not feel as costly in monetary terms because it holds the added benefit to the employee’s self-evaluation. This explanation is beyond the scope of the current research, and future studies could shed more light on this topic.

Another important finding, which is of particular relevance to the dark side of OCB in terms of the organization, is that professional costs (time taken from other clients, reduced in-role performance) were mentioned less than most other costs. This suggests that, at least for some occupations, going beyond the call of duty may increase customer well-being and satisfaction in the short term. However, it might also result in a reduced level of well-being both in the short and long term. As such, it can damage the organization’s functioning as a whole.

Lastly, we looked for differences between occupational groups in terms of both OCBCs and their associated costs. Interestingly, even though the three examined groups (education, health and welfare) vary in many aspects, such as characteristics of the profession, type of clients and working conditions, the qualitative analysis found more similarities than differences. Cross-coding analysis did allow us to delve deeper and identify some variations between occupational domains and professions, both in terms of the type of informal resources provided and in terms of costs to employees, yet on the whole similarities between occupations were much more apparent. This lack of significant differences might be attributed to the growing need of workers in many occupational domains to help their organizations remain competitive with fewer formal resources – in short, to “do more with less” (e.g., Simons and Mason, 2003). This is linked to concern for the organization’s ability to function properly in the long term.

### Theoretical Contribution

The main theoretical contribution of the current paper is in delineating the types of informal resources that are invested in customers as part of OCBCs, as well as the costs associated with them for employees. These costs can be expected to differ from costs associated with other types of OCB (e.g., Bolino and Turnley, 2005), because employees are likely to provide different types of aid to customers in great need than to their colleagues (i.e., OCBI). Highlighting the unique resources invested as OCBC and their associated costs is important and timely because OCBCs are increasingly more prevalent, as part of a growing trend to “do more with less.”

Research has shown a positive correlation between OCBC and other types of OCB (OCBI and OCBO), indicating that individuals who tend to go beyond the call of duty do so in all domains (e.g., Ma and Qu, 2011). However, meta-analytical evidence suggests that individuals who engage in more OCBs experience less work interference with family (Amstad et al., 2011). The results from the current study suggest otherwise: they show OCBCs to be associated with a variety of costs, including increased interference with family.

This discrepancy between past and current findings can be explained in a number of ways. First, the reported meta-analytical findings may simply indicate that individuals who are prosocial and proactive will manifest these tendencies in most or all their roles (e.g., good performers on the job, helpful coworkers, engaged family members), but this does not mean there are no costs associated with going the extra mile. The use of a qualitative method in the current study may have enabled us to uncover costs that are otherwise hard to detect. The main advantage of using a qualitative method is the ability to delve deeply into participants’ daily experience.

Another possibility is that other OCB types that are more readily observable and acknowledged indeed have an energizing effect that can result in an overall increase in the employee’s resource pool. In contrast, OCBCs – which often go unnoticed and are part of the global trend to “do more with less” – may be costlier or have fewer benefits for employees. A third explanation is that the types of costs associated with OCBCs are different than those associated with OCBI and OCBO, presenting a bigger role conflict and infringing more on family life. A major characteristic of the OCBCs revealed in the current study is the worker’s overall extra investment of time and energy in clients, often outside of work hours. One implication of this engagement is their continuous and never-ending preoccupation with work-related matters. In other words, if employees are constantly “at work” even when at home, then the interference of work with the non-work domain is inevitable.

The surprising dearth of reported professional costs of OCBCs may also shed light on work interference with family life. One can assume that the vast engagement of workers in OCBCs unavoidably results in a decrease in their ability to perform well in their formal service delivery. Yet, such a cost was hardly reported, in contrast to the most common reported cost of interference with children and partners. This suggests that when employees find themselves in situations where they have to provide their own resources in order to deliver adequate service to customers, they generally do not allow that to negatively influence other customers. As argued in Resource Depletion theory (Rothbard, 2001), individuals have a finite amount of physical and psychological resources. When more resources are invested in the work domain, a deficiency of resources in the non-work domain is created. This might have specific implications for women, due to their primary caretaker role within the family and the female majority in caring and social professions (Lavee and Strier, 2019). Our findings join literature which argues that women workers, especially those employed in social services, tend to conform to social role expectations (Riccucci, 2017) that they be nurturing, patient and able to sense and respond to overall client needs (Guy and Newman, 2004). Women tend to act in keeping with these expectations in order to avoid negative consequences (Potipiroon et al., 2019).

These results resemble findings from the work-stress literature, whereby employees who are confronted with organizational constraints (i.e., things that get in the way of getting the job done), such as fewer resources, experience decreased well-being and increased work–family conflict (Pindek and Spector, 2016). However, these constraints do not seem to negatively affect in-role performance, as employees find ways to get the work done despite the limited resources or interferences (Pindek et al., 2015).

### Practical Implications

Our findings suggest that service providers who engage in high levels of OCBCs experience depleted resources and higher strain levels, which can have negative long-term effects on their productivity (Dewa et al., 2014). Therefore, organizations that want to keep their employees productive over time need to acknowledge the gap between the formal resources currently provided by the organization and the actual resources needed to perform well – that is, to provide quality customer service. Interventions aimed at reducing this gap can have various benefits, including increased productivity and customer satisfaction while maintaining employees’ health and well-being over time. Other interventions can target the costs themselves. For example, finding a way to compensate employees who invest their personal resources (e.g., time, money, and effort) may help them regain some resources in lieu of those they have invested or lost. Feeling that their efforts are acknowledged may help counteract impediments to their motivation, well-being and productivity.

### Limitations and Future Research

While qualitative research is highly useful for an in-depth understanding of individual experience, it does not allow for systematic comparisons. Furthermore, owing to the typically low number of participants and their homogeneity, generalizability is limited. Nonetheless, the sample in the current study was relatively large and diverse in terms of occupations, seniority and ages (but not gender, as discussed below), which renders our results somewhat more generalizable than other qualitative studies that typically have smaller and less diverse samples, at least when discussing similar occupational groups.

A related issue is that the current study focused mostly on professional service providers, as opposed to non-professional employees who provide customer service (Thakor and Kumar, 2000). Results from the current study may not replicate in full in non-professional customer service samples (e.g., employees in customer service centers). Professional workers more often define themselves in terms of their profession and see it as a reason for having to provide informal resources, stating that their position as teacher or social worker, doctor or nurse, requires them to always have to go the extra mile. Future research could examine non-professional customer service employees and compare the types of informal resources and associated costs with professional customer service employees. Comparisons between the private and public sectors may also uncover differences in the provision of informal resources.

A related point deserving mention is that we were not able to detect meaningful differences between occupational groups in the types of informal resources provided or the associated costs of OCBCs. This does not necessarily reflect a real lack of difference; it is possible that a finer lens needs to be used, looking at specific occupations rather than occupational groups. Another option is to link specific contextual factors (e.g., workload) within the service providers’ jobs with the types of informal resources and OCBC costs, as part of a future quantitative investigation. The use of experience sampling methodology could help uncover the frequency with which each type of informal resource is provided.

Another limitation is that the vast majority of research participants were women. This gender distribution, although not initially intended, is consistent with the general overrepresentation of women in the education, health and welfare professions. Previous research indeed indicated that women perform differently than men in terms of service provision (e.g., Guy and Newman, 2004), and gender differences in OCB performance in general have been detected (e.g., Kidder, 2002). In light of the overrepresentation of women in the current research, additional studies could benefit from including more male participants and exploring the possible gendered nature of engagement in OCBCs.

Lastly, one of the most surprising findings was the relatively low professional cost reported by participants, even though we expected employees’ vast investment in OCBC for certain customers to interfere with some aspect of their in-role performance, such as providing adequate formal service to other customers. Future research may triangulate employees’ subjective perceptions regarding their formal work performance with other more objective indicators, such as supervisors’ reports of employees’ performance, to better capture the organizational costs of OCBCs.

## Conclusion

The general trend to “do more with less” is associated with service employees investing increasingly more informal resources in their customers as part of their desire to provide quality service. As such, highlighting the possible “dark side” of this phenomenon is particularly important. The current study shows that, on the whole, customer service employees invest their personal resources in order to help customers at great costs to their work and non-work lives.

## Data Availability Statement

The raw data supporting the conclusions of this article will be made available by the authors, without undue reservation, to any qualified researcher.

## Ethics Statement

The studies involving human participants were reviewed and approved by University of Haifa, Faculty of Social Welfare and Health Sciences Ethics Review Board. Written informed consent for participation was not required for this study in accordance with the national legislation and the institutional requirements.

## Author Contributions

EL was in charge of collecting and analyzing the data. SP was in charge of writing much of the manuscript. EL and SP both contributed to the idea development and conceptualization.

## Conflict of Interest

The authors declare that the research was conducted in the absence of any commercial or financial relationships that could be construed as a potential conflict of interest.
